# Enhancement of Electrical Conductivity of Aluminum-Based Nanocomposite Produced by Spark Plasma Sintering

**DOI:** 10.3390/nano11051150

**Published:** 2021-04-28

**Authors:** Nicolás A. Ulloa-Castillo, Roberto Hernández-Maya, Jorge Islas-Urbano, Oscar Martínez-Romero, Emmanuel Segura-Cárdenas, Alex Elías-Zúñiga

**Affiliations:** 1 Tecnologico de Monterrey, Department of Mechanical Engineering and Advanced Materials, School of Engineering and Sciences, Av. Eugenio Garza Sada Sur 2501, Monterrey 64849, Nuevo León, Mexico; nicolas.ulloa@tec.mx (N.A.U.-C.); jor_isur@tec.mx (J.I.-U.); esca@tec.mx (E.S.-C.); 2 Siemens, Research and Development Department, Libramiento Arco Vial Poniente Km 4.2, Santa Catarina 66350, Nuevo León, Mexico; roberto.hernandez_maya@siemens.com

**Keywords:** aluminum matrix nanocomposites, multi-walled CNTs, electrical conductivity, spark plasma sintering

## Abstract

This article focuses on exploring how the electrical conductivity and densification properties of metallic samples made from aluminum (Al) powders reinforced with 0.5 wt % concentration of multi-walled carbon nanotubes (MWCNTs) and consolidated through spark plasma sintering (SPS) process are affected by the carbon nanotubes dispersion and the Al particles morphology. Experimental characterization tests performed by scanning electron microscopy (SEM) and by energy dispersive spectroscopy (EDS) show that the MWCNTs were uniformly ball-milled and dispersed in the Al surface particles, and undesirable phases were not observed in X-ray diffraction measurements. Furthermore, high densification parts and an improvement of about 40% in the electrical conductivity values were confirmed via experimental tests performed on the produced sintered samples. These results elucidate that modifying the powder morphology using the ball-milling technique to bond carbon nanotubes into the Al surface particles aids the ability to obtain highly dense parts with increasing electrical conductivity properties.

## 1. Introduction

Carbon-based nanostructures have been widely used for the preparation and processing of metal composites for improving their physical and mechanical properties [1,2,3]. In this sense, aluminum (Al)-based nanocomposites have proven to be excellent materials for the production of parts with enhanced properties for the aerospace and automobile industries [4,5,6]. Getting uniform reinforcement dispersion becomes one of the main challenges when preparing nanocomposites, which in principle, promotes a better interaction between the particle powders in the matrix [7], resulting in a high densification behavior after the powder consolidation.

Multi-walled or single-walled CNTs (MWCNTs and SWCNTs, respectively) are ideal nanostructures to reinforce Al-based materials [1,8] because of their excellent physical properties [9,10,11]. It is well-known that the Young modulus value for CNTs is about 1 TPa [12], while the thermal expansion coefficient can reach values close to $2\times 10^{-5}$ K^−1^ [13]. However, a relevant issue of using such nanostructures is the Van der Waals force interactions, which can be estimated considering the diameters and number of walls [14] since CNTs clusters and agglomerations lead to the formation of pores or voids during the nanocomposite powder consolidation [15]. To overcome these limitations, some solution-assisted methodologies that promote better interaction between Al powder and CNTs, that allows CNT uniform dispersion and avoiding agglomerations have been proposed [16,17]. These strategies involve the use of oxidative reagents, which could induce some considerable damage in the CNT structure [18] and hence, hindering their intrinsic properties. Another feasible solution is related to high-energy ball-milling technique widely used for the preparation of materials in powder metallurgy [2,19,20]. The latter due to its capability of achieving uniform dispersion of the reinforcement while promoting interfacial bonding between the metallic matrix and the added filler, reducing possible damage in the reinforcement structure while keeping its physical properties [13,14,15,16]. Additionally, ball-milling is capable of modifying the powder morphology which favors substantially the powder particle interaction and thus, impacting positively the consolidation process for getting high densification samples [21].

On the other hand, there are several experimental routes for consolidation of Al-based nanocomposite powders, like the ones reported for hot extrusion [22], hot rolling [23], and friction stir processes [24,25], to name a few. Among these, spark plasma sintering (SPS) is a process that provides high densification produced parts, controlling grain growth kinetics, improving the bonding in the grain boundaries, optimizing energy consumption and, saving production time and cost [22,26,27]. Some authors have identified and discussed in the literature experimental factors that could affect the SPS process of nanocomposites. For instance, it was investigated in [28,29,30] the impact of the CNT content in the consolidation of nanocomposite powders when varying experimental conditions like load pressure, temperature and holding sintering time. There, the authors concluded that the formation of large-sized CNT clusters hinder particle interaction during sintering process leading to the formation of cracks, pores or voids [1,31]. These results have allowed to identify the best conditions to develop Al-based nanocomposites with enhance physical properties. Other research works focus on studying how the wt % concentration of CNT influences the sample densification [21] and dispersion to create an effective 2D CNT network capable of improving electrical conductivity [25,31,32,33].

Therefore, the article aims consist in investigating the effect of adding 0.5 wt % concentration of MWCNT embedded into Al powder through ball-milling technique. Three nanocomposite samples were produced via spark plasma sintering (SPS) process and then, these fabricated samples were subjected to experimental characterizations to evaluate their MWCNTs distribution, densification, and electrical conductivity properties. The findings of this research work elucidate the steps needed to scale up industrial Al-based nanocomposites for production of engineering components.

## 2. Materials and Methods

Aluminum powders with a purity grade >99% was purchased from Ampal Inc., Palmerton, PA, USA. The industrial-grade MWCNTs (carbon content >90%) with outer diameters ranging from 50–80 nm, inner diameters ranging from 5–15 nm and lengths between 10–20 μm were purchased from Nanostructured & Amorphous Materials, Inc., Houston, TX, USA. All materials were used as received.

### 2.1. Al-Based MWCNTs Nanocomposite Powder Preparation

MWCNTs concentration of 0.5 wt % were added into the Al powders and processed through a planetary ball-mill equipment (Retsch, PM 100, purchased from Advanced Analytical Systems, Guadalajara, Jalisco, Mexico) using stainless-steel balls with 2 mm of diameter. Here, a ball-to-powder ratio of 10:1 with milling speed of 225 rpm during 30 min were set to process 200 grs of aluminum nanocomposite powder needed to produce three Al-based MWCNTs specimens.

### 2.2. Sintering Consolidation of the Al-Based MWCNTs Specimens

To produce the material samples using the Al-MWCNTs powders, spark plasma sintering (SPS) equipment was used (Dr. Sinter SPS-1050 equipment purchased from Fiju Electronic Industrial, Tsurugashima, Saitama, Japan) following procedure steps reported in [34]. A graphite die with 40 mm of diameter and 12.7 mm of wall thickness dimensions was loaded with the nanocomposite powder and placed into the SPS vacuum chamber. Then, the powders were consolidated applying compaction pressure of 50 MPa using graphite punches. The sintering process was performed at the temperature of 620 °C at the heating rate of 50 °C/min for 5 min with operating conditions similar to the ones reported in [33]. Finally, the sintered disk-shaped samples with 40 mm in diameter and 10 mm of thickness were released at the die room temperature. In total, three samples, named M1, M2 and M3, were produced using the same MWCNT content (0.5 wt%) and similar sintering conditions.

### 2.3. Scanning Electron Microscopy (SEM)

To investigate the cross-sectional surface morphology of the Al-based MWCNTs nanocomposites, SEM (ZEISS, EVO MA 25, Oberkochen, Germany) microscope experimental measurements were performed. The system was operated at 20 kV as accelerating voltage considering 7.5 mm of work distance. The corresponding analyses were performed by taking secondary electrons (SE) and backscattered electrons (BSE) images to investigate the morphology and the chemical composition, respectively. The MWCNTs distribution was investigated by analyzing energy dispersive spectroscopy (EDS) elemental mapping images.

### 2.4. Densification

To measure the density of the sintered material samples, Archimedes’ principle is used with deionized water as the immersion medium in accordance with ASTM B962–17 norm. Thus, the experimental density values can be determined using the Equation:

(1) $$\rho_{e}=\frac{A\;\rho_{w}}{A-\left(B-C\right)},$$

where $\rho_{e}$ is the sample density, *A* is the sample mass, *B* is the oil-impregnated sample mass, *C* is the oil-impregnated sample mass immersed in water, and $\rho_{w}$ is the density of water (0.9956 g/cm^3^). The relative density, $\rho_{r},$ of the nanocomposites is determined using:

(2) $$\rho_{r}=\frac{\rho_{e}}{\rho_{c}}\times 100,$$

where $\rho_{C}$ is the theoretical density which can be assessed by considering the rule of mixtures $\rho_{C}=\rho_{CNT}V_{CNT}+\rho_{Al}V_{Al}$, where $\rho_{CNT}$ and $\rho_{Al}$ are the theoretical carbon nanotubes and aluminum densities, respectively, *V* is the volume fraction of either, the MWCNT (2.1 g/cm^3^) filler or the Al (2.7 g/cm^3^) matrix.

### 2.5. Tomography

The porosity distribution within the Al-based MWCNTs samples was carried out through an X-ray tomography equipment (Nikon, XT H 225 ST—Industrial CT Scanning, Brighton, MI, USA). The images were taken in a region close to the center of the samples. Then, the sample total porosity content can be found from the following Equation:

(3) $$Porosity\;\left(\%\right)\;=100\%-\rho_{r}.$$

### 2.6. X-ray Diffraction (XRD) Measuremrnts

The sintered material samples experimental characterizations were performed using a XRD system (Malvern PanAnalytical, X’Pert Pro PW1800, UK) operated at 45 mA and 40 kV. The diffractograms were measured using the Bragg–Brentano geometry in reflection mode with a copper Kα tube in the 2θ range of 10°–145° with a scanning rate of 2°/min. The crystallite size was found using the modified Williamson–Hall equation [34]

(4) $$\beta_{hkl}cos\theta =\frac{K\lambda }{d}+4\varepsilon \sin\theta ,\;\mathrm{with}\;\beta_{hkl}=\sqrt{{\left(\beta_{hkl}\right)}_{\mathrm{measured}}^{2}+{\left(\beta_{hkl}\right)}_{\mathrm{instrumental}}^{2}}$$

where $\beta_{hkl}=\sqrt{{\left(\beta_{hkl}\right)}_{\mathrm{measured}}^{2}+{\left(\beta_{hkl}\right)}_{\mathrm{instrumental}}^{2}}$ and $\;{\left(\beta_{hkl}\right)}_{\mathrm{measured}}$ is the measured value of the full width half maxima of the maximum intensity peak (peak position θ), and ${\left(\beta_{hkl}\right)}_{\mathrm{instrumental}}$ is the standard sample (strain free annealed pure Al sample), *K* is a constant, λ is X-ray wavelength, and ε represents the average lattice strain. On the other hand, the dislocation density ($\rho_{d}$) can be estimated using ε value and crystallite size value, *d*, can be found using the following Equation [29,35]:

(5) $$\rho_{d}=\frac{2\sqrt{3}{\left(\varepsilon^{2}\right)}^{\frac{1}{2}}}{bd}$$

where *b* is the Burgers vector (0.286 nm) [30,36].

### 2.7. Electrical Conductivity Tests

To measure the electrical conductivity of the produced material samples, we performed experimental measurements using a Sigmascope (model SPM10, purchased from Fisher, Monterrey, Nuevo León, México) equipment, whose operation is based on eddy current methodology and in accordance with the ASTM E 1004 and DIN EN 2004-1 norms. The corresponding values were collected from 4 different zones of the nominal cross-section area of each sintered sample and considering an average of 10 measurements per zone. The electrical resistivity, ρ in Ohms meter, was determined using the equation:

(6) $$\rho =\frac{1}{\sigma }$$

where σ is the electrical conductivity in Siemens per meter.

## 3. Results and Discussion

### 3.1. Cross-Sectional Morphology and EDS Elemental Mapping Analysis

**Figure 1:**
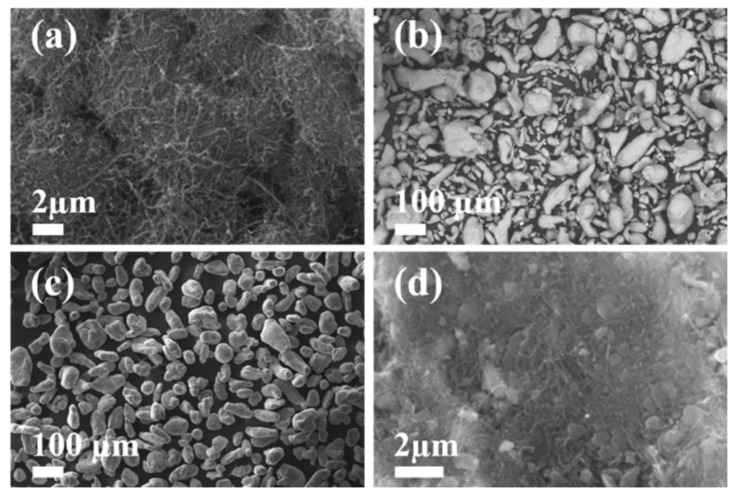
SE-SEM micrographs for: (**a**) highly entangled and agglomerated as-received MWCNTs; (**b**) as-received Al powder; (**c**) uniform size distribution of ball-milled Al-MWCNTs nanocomposite powders; (**d**) MWCNTs dispersion within the ball-milled Al powder surface.

The surface morphology of the purchased Al powders and of the pristine MWCNTs are shown in Figure 1a,b. The SE-SEM micrograph for the as-received MWCNTs show the presence of clusters and agglomerations of CNT entangled caused, mainly, by the strong CNT-CNT Van der Waals forces [37]. In the case of the as-received Al virgin powder, the corresponding SE-SEM image show elongated particle powders with irregular morphology. Figure 1c shows the influence of the ball-milling process in reducing the powders particle size and how the smallest ones were agglomerated to form bigger particles, resulting, in general, in a homogenization in ball-milled powder morphology. The zoom-in view of Figure 1d confirms good MWCNTs dispersion embedded into the Al powder surface.

**Figure 2:**
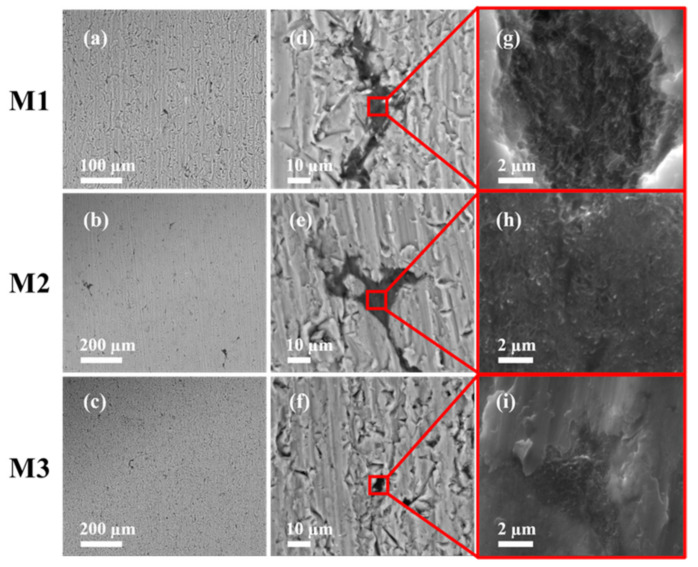
Cross-sectional BSE-SEM (**a**–**c**) for sintered Al-based MWCNTs nanocomposites M1, M2 and M3. Despite the good dispersion of the MWCNTs, images (**d**–**f**) show some MWCNTs agglomerations along the samples cross section. SE-SEM micrographs (**g**–**i**) confirm the appearance of MWCNTs clusters which is an indication that carbon nanotubes dispersion was not good.

Figure 2 shows the cross-section images of the sintered samples M1, M2 and M3. Figure 2a–c shows the formation of similar microstructure in all sintered samples. These BSE-SEM micrographs exhibit the appearance of small fractures that could be caused during the samples cross-sectional cut. Figure 2d–f shows a zoom-in view of the samples microstructure in which some micro-pores are observed. Furthermore, the dark areas in the surrounding of the Al grains correspond to the MWCNTs. This conclusion is confirmed via the zoom-in views shown in Figure 2g,h,i.

**Figure 3:**
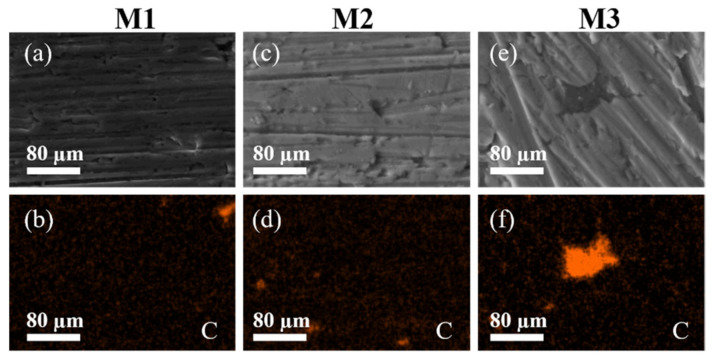
BSE-SEM micrographs (**a**,**c**,**e**) confirm that the MWCNTs are mainly located in the surrounding of the Al grains creating a CNT network within the SPS material samples. Images (**b**,**d**,**f**) are the samples M1, M2 and M3 energy dispersive spectroscopy (EDS) elemental mapping. The MWCNT distribution is similar in all sintered samples. Image (**f**) shows a cluster of MWCNT.

Figure 3 shows experimental images of the MWCNT dispersion obtained via EDS-SEM elemental mapping for Al and C content in all sintered nanocomposites. Figure 3b,d,f illustrates the tracking performed for C (orange). These images exhibit few spots of CNTs agglomerations which is an indication of the uniform distribution of MWCNTs along the samples cross-section. The elemental mapping shown in Figure 3a,c,e confirms that the MWCNTs are mainly located in the surrounding of the Al grains creating a CNT network within the sintered samples. Moreover, the latter findings indicate that the experimental characterization measurements performed in the SPS samples can help to identify if the MWCNTs are well dispersed.

### 3.2. Crystallite Size of Sintered Al-Based MWCNT Nanocomposites

**Figure 4:**
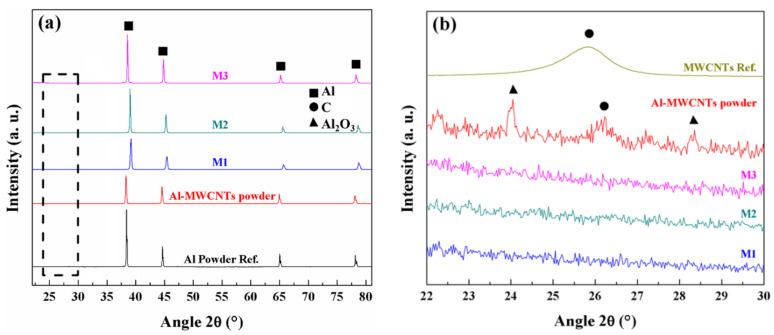
(**a**) XRD diffractograms for Al-MWCNTs powder and for the sintered Al-based nanocomposites M1, M2, and M3 samples, and the characteristic reflection planes for Al powder. (**b**) Zoom-in view of the Al-based nanocomposite M1, M2, and M3 samples showing the characteristic (002) graphitic plane. The MWCNTs and Al diffractograms were included and scaled for comparison purposes.

Figure 4 shows XRD experimental characterization results obtained for the nanocomposite powders and the sintered samples. Figure 4a illustrates the samples M1, M2 and M3 diffractograms compared with the Al-MWCNT nanocomposite powder and with the Al powder reference. It is also observed the characteristic peaks of the Al unit cell at 38.4° and 44.6°, that corresponds to the (111) and (200) planes, respectively. One can notice that the peaks related to Al for samples M1-M3 are shifted, in comparison to the Al powder reference, as a result of the stresses induced during the sintering of the nanocomposite powders.

Figure 4b shows a zoom-in view in the region in which the signal of the graphitic structures can be detected, commonly around 26° which agrees with the (002) plane (interplanar spacing of 0.34 nm). Note that the diffractograms for M1, M2 and M3 show no presence of MWCNTs signal, while the diffractogram for the Al-MWCNT nanocomposite powder show a small peak around the angle expected for the MWCNT signal. The latter is associated to the relatively low wt % concentration and uniform dispersion of the MWCNTs added into the aluminum powders. The corresponding experimental diffractogram for the industrial-grade MWCNTs was included and scaled for comparison purposes. The small peaks observed around 22° and 29° in all sintered samples are attributed to Al_2_O_3_ formed during the spark plasma sintering process.

Table 1 summarizes the crystallite size of the sintered nanocomposites computed from Equation (4). Notice that the crystallite size of the as-received Al powder reduce from 404 nm to 30 nm after the Al-MWCNTs powder is ball-milled. Upon sintering the ball-milled nanocomposite powders, crystallite size values slightly increase to 48, 40, and 34 nm for samples M1, M2, and M3, respectively. The small variations in the crystallite size values obtained after the SPS consolidation of the ball-milled powders corroborate fabrication samples repeatability. The crystallite size variation among samples is mainly due to the uniform dispersion of MWCNTs within the Al matrix, that in addition to the experimental sintering conditions, allows to attach the CNTs in the boundaries of the Al grain and thus, limiting their growth. In [30], Singh et al. found similar results in their Al-MWCNTs (0.5 wt %) nanocomposite sintered with similar SPS experimental conditions. They concluded that the resulting Al grain can be affected by: (a) the creation of dislocations caused by plastic deformation during ball-milling process, (b) sub-grain formation during samples sintering processes, and (c) recrystallization processes which impact directly on the crystallite size. The dislocation density was found to increase from $1.79\times 10^{13}$ m^−2^ (as-received Al) to $3.60\times 10^{14}$ m^−2^ for the Al-MWCNTs composite powder.

### 3.3. Densification Behavior and Porosity of Sintered Al-Based MWCNT Nanocomposites

The relative densities and porosity percentage values calculated using Equation (3) for the sintered samples are summarized in Table 1. High densification values obtained from the sintered nanocomposites could be attributed among other factors, to the uniform MWCNTs dispersion, avoiding the formation of large-sized agglomerations and thus, preserving an effective particle interaction during the SPS process. Despite the increase of dislocation density value calculated for milled nanocomposite powder, which can hinder the densification process via the formation of nanocrystalline regions [29], the morphology homogenization of powder particles promotes particle interaction during the sintering. In this sense, it is well-known that the MWCNTs act as a barrier to the Al particle rearrangement, hindering the densification process and creating high porosity density in the sintered sample, especially for nanocomposites with high MWCNTs content. The appearance of micro-pores or small cracks induced by grain dislocations during the sintering process [30] does not allow to achieve samples full densification as confirmed by SEM images shown in Figure 2. Despite these negative effects, the consolidation and densification of the SPS samples reach higher percentage values above 98%, as listed in Table 1.

**Figure 5:**
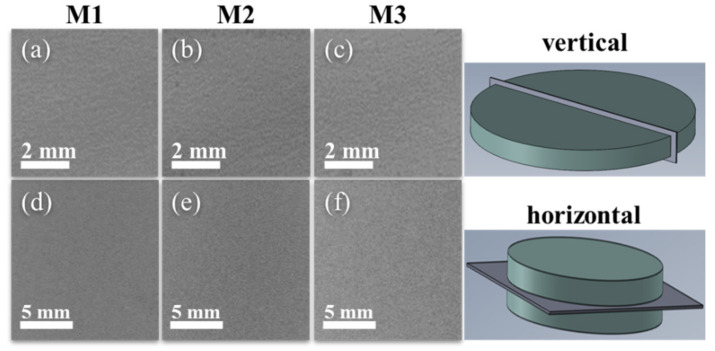
X-ray tomographies of (**a**–**c**) vertical slices to obtain a side view, and (**d**–**f**) horizontal slices to obtain a top view of the sintered nanocomposites. Both set of tomographies were taken in an area close to the center and show homogenous consolidation without the presence of large-sized pores.

Figure 5 shows X-ray tomography images taken in vertical (perpendicular to the base) and horizontal (parallel to the base) configurations in the region close to the center of the sintered nanocomposites that were used to explore pore formation. As shown in Figure 5, the appearance of large-sized pores is not evident.

### 3.4. Electrical Conductivity

The electrical conductivity of the Al-based nanocomposites was measured in three samples zones to evaluate CNTs homogeneous dispersion within the samples: zone 1 (left sample end), zone 2 (middle), and zone 3 (right sample end). The recorded values are listed in Table 2. The average value obtained in all sintered samples is around 24.1 MS/m which corresponds to a 40% in the conversion of the International Annealed Copper Standard (IACS). The homogenous CNT dispersion achieved during the ball-milling promoted the formation of an effective network within the Al matrix so that the MWCNTs were strategically located at the surroundings of the Al grains. Such condition was key to promote an efficient carrier electron transport induced by tunneling effect and percolation threshold phenomena [38,39].

In a previous author’s work [33], the electrical value measured for Al-based nanocomposite reinforced with 0.5 wt % concentration of MWCNTs was around 26.1 MS/m (45.1% IACS), which is 5% higher than the value of 24.1 MS/m found in samples M1, M2, and M3. The difference between electrical conductivity values could be due to the usage of Al and MWCNTs industrial grade materials (as received), to the alumina formed during the ball-milling dispersion of the MWCNTs (see Figure 4b), that acts as a barrier between the MWCNTs and Al, and to the sintering conditions applied for the powder consolidation, that could affect the electrical performance of the nanocomposites, as found by Zhang et al. [25], and Chen and Kondoh [40].

## 4. Conclusions

In this paper, the electrical conductivity and densification properties of samples fabricated via the SPS process using Al powder reinforced with 0.5 wt % of MWCNTs have been investigated. One can conclude from the experimental characterizations and electrical conductivity measurements that:

- Ball-milling process modifies Al particle morphology and size enhancing powder particle interaction and MWCNTs bonding that allows to produce high densification SPS process samples.
- A good dispersion of MWCNTs along the produced samples is confirmed by EDS elemental mapping images.
- After the fabrication of the composite samples M1, M2, and M3 via SPS process, the crystallite size of the ball-milled Al-MWCNTs powders changes from 30 nm to an average value of 43 nm, which is an indication of the MWCNTs bonding into the boundaries of the aluminum grains.
- The electrical conductivity value, in all sintered samples, improves around 40% IACS which is attributed to the effective multi-wall-carbon-nanotubes network formed within the nanocomposite material, that promotes electron transport.

Finally, our research methodology and findings elucidate the steps that one must follow in order to use, for a mass production of metallic parts, Al-based nanocomposite powder to enhance end parts electrical conductivity properties.

## Figures and Tables

**Table 1:** Crystallite size, relative density and porosity percentage calculated for samples M1, M2, M3, Al powder, and ball-milled Al-MWCNTs powder.

Sample	Description	Crystallite Size (nm)	Relative Density (%)	Porosity (%)
Al Powder Ref.	As received	404	-	-
Al-MWCNTs Powder	Ball-milled	30	-	-
M1	Al+MWCNTs (0.5 wt %)	48	98.2	1.72
M2	Al+MWCNTs (0.5 wt %)	40	99.7	0.23
M3	Al+MWCNTs (0.5wt %)	34	98.8	1.16

**Table 2:** Electrical conductivity and electrical resistivity values measured from three different zones of each sintered Al-based nanocomposite, as well as their corresponding IACS values.

Sample	Resistivity (nΩ-m)			Conductivity (MS/m)			IACS (%)		
	Zone 1	Zone 2	Zone 3	Zone 1	Zone 2	Zone 3	Zone 1	Zone 2	Zone 3
M1	41.2	41.2	40.6	24.2	24.2	24.6	41.8	41.8	42.4
M2	41.5	41.3	41.4	24.0	24.1	24.1	41.5	41.7	41.6
M3	42.3	42.3	41.4	23.6	24.2	24.1	40.7	41.8	41.6

## References

[1] Mohammed S.M.A.K., Chen D.L. (2020). Carbon Nanotube-Reinforced Aluminum Matrix Composites. Adv. Eng. Mater..

[2] Veillère A., Kurita H., Kawasaki A., Lu Y., Heintz J.-M., Silvain J.-F. (2019). Aluminum/Carbon Composites Materials Fabricated by the Powder Metallurgy Process. Materials.

[3] Kumar Sharma A., Bhandari R., Aherwar A., Rimašauskienė R., Pinca-Bretotean C. (2020). A study of advancement in application opportunities of aluminum metal matrix composites. Mater. Today Proc..

[4] Bradbury C.R., Gomon J.K., Kollo L., Kwon H., Leparoux M. (2014). Hardness of Multi Wall Carbon Nanotubes reinforced aluminium matrix composites. J. Alloys Compd..

[5] Singla D., Amulya K., Murtaza Q. (2015). CNT Reinforced Aluminium Matrix Composite—A Review. Materials Today: Proceedings.

[6] Mavhungu S.T., Akinlabi E.T., Onitiri M.A., Varachia F.M. (2017). Aluminum Matrix Composites for Industrial Use: Advances and Trends. Procedia Manuf..

[7] Liu Z.Y., Xu S.J., Xiao B.L., Xue P., Wang W.G., Ma Z.Y. (2012). Effect of ball-milling time on mechanical properties of carbon nanotubes reinforced aluminum matrix composites. Compos. Part A Appl. Sci. Manuf..

[8] Sajeeb Rahiman A.H., Robinson Smart D.S., Antony K., Davim J.P. (2018). Aluminium Carbon Nanotube Composites—A Review on Latest Approaches.

[9] Ajayan P.M., Ebbesen T.W. (1997). Nanometre-size tubes of carbon. Rep. Prog. Phys..

[10] Dai H. (2002). Carbon nanotubes: Opportunities and challenges. Surf. Sci..

[11] De Volder M.F.L., Tawfick S.H., Baughman R.H., Hart A.J. (2013). Carbon Nanotubes: Present and Future Commercial Applications. Science.

[12] Xiao J.R., Gama B.A., Gillespie J.W. (2005). An analytical molecular structural mechanics model for the mechanical properties of carbon nanotubes. Int. J. Solids Struct..

[13] Deng L., Young R.J., Kinloch I.A., Sun R., Zhang G., Noé L., Monthioux M. (2014). Coefficient of thermal expansion of carbon nanotubes measured by Raman spectroscopy. Appl. Phys. Lett..

[14] Esawi A.M.K., Morsi K., Sayed A., Taher M., Lanka S. (2011). The influence of carbon nanotube (CNT) morphology and diameter on the processing and properties of CNT-reinforced aluminium composites. Compos. Part A Appl. Sci. Manuf..

[15] Tjong S.C. (2013). Recent progress in the development and properties of novel metal matrix nanocomposites reinforced with carbon nanotubes and graphene nanosheets. Mater. Sci. Eng. R Rep..

[16] Liu Z.Y., Zhao K., Xiao B.L., Wang W.G., Ma Z.Y. (2016). Fabrication of CNT/Al composites with low damage to CNTs by a novel solution-assisted wet mixing combined with powder metallurgy processing. Mater. Des..

[17] Fan G., Jiang Y., Tan Z., Guo Q., Xiong D.-b., Su Y., Lin R., Hu L., Li Z., Zhang D. (2018). Enhanced interfacial bonding and mechanical properties in CNT/Al composites fabricated by flake powder metallurgy. Carbon N. Y..

[18] Datsyuk V., Kalyva M., Papagelis K., Parthenios J., Tasis D., Siokou A., Kallitsis I., Galiotis C. (2008). Chemical oxidation of multiwalled carbon nanotubes. Carbon N. Y..

[19] Sharma P., Sharma S., Khanduja D. (2015). On the Use of Ball Milling for the Production of Ceramic Powders. Mater. Manuf. Process..

[20] Morsi K., Esawi A.M.K., Lanka S., Sayed A., Taher M. (2010). Spark plasma extrusion (SPE) of ball-milled aluminum and carbon nanotube reinforced aluminum composite powders. Compos. Part A Appl. Sci. Manuf..

[21] Saheb N., Iqbal Z., Khalil A., Hakeem A.S., Al Aqeeli N., Laoui T., Al-Qutub A., Kirchner R. (2012). Spark plasma sintering of metals and metal matrix nanocomposites: A review. J. Nanomater..

[22] Choi H.J., Kwon G.B., Lee G.Y., Bae D.H. (2008). Reinforcement with carbon nanotubes in aluminum matrix composites. Scr. Mater..

[23] Choi H., Shin J., Min B., Park J., Bae D. (2009). Reinforcing effects of carbon nanotubes in structural aluminum matrix nanocomposites. J. Mater. Res..

[24] Liu Z.Y., Xiao B.L., Wang W.G., Ma Z.Y. (2014). Tensile strength and electrical conductivity of carbon nanotube reinforced aluminum matrix composites fabricated by powder metallurgy combined with friction stir processing. J. Mater. Sci. Technol..

[25] Zhang S., Chen G., Qu T., Wei J., Yan Y., Liu Q., Zhou M., Zhang G., Zhou Z., Gao H. (2021). A novel aluminum-carbon nanotubes nanocomposite with doubled strength and preserved electrical conductivity. Nano Res..

[26] Ujah C.O., Popoola A.P.I., Popoola O.M., Aigbodion V.S. (2019). Optimisation of spark plasma sintering parameters of Al-CNTs-Nb nano-composite using Taguchi Design of Experiment. Int. J. Adv. Manuf. Technol..

[27] Viswanathan V., Laha T., Balani K., Agarwal A., Seal S. (2006). Challenges and advances in nanocomposite processing techniques. Mater. Sci. Eng. R Rep..

[28] Singh L.K., Bhadauria A., Jana S., Laha T. (2018). Effect of Sintering Temperature and Heating Rate on Crystallite Size, Densification Behaviour and Mechanical Properties of Al-MWCNT Nanocomposite Consolidated via Spark Plasma Sintering. Acta Metall. Sin. (Engl. Lett.).

[29] Singh L.K., Bhadauria A., Oraon A., Laha T. (2019). Spark plasma sintered Al-0.5 wt% MWCNT nanocomposite: Effect of sintering pressure on the densification behavior and multi-scale mechanical properties. Diam. Relat. Mater..

[30] Singh L.K., Bhadauria A., Laha T. (2019). Al-MWCNT nanocomposite synthesized via spark plasma sintering: Effect of powder milling and reinforcement addition on sintering kinetics and mechanical properties. J. Mater. Res. Technol..

[31] Ujah C.O., Popoola A.P.I., Popoola O.M., Aigbodion V.S. (2020). Influence of CNTs addition on the mechanical, microstructural, and corrosion properties of Al alloy using spark plasma sintering technique. Int. J. Adv. Manuf. Technol..

[32] Xu C.L., Wei B.Q., Ma R.Z., Liang J., Ma X.K., Wu D.H. (1999). Fabrication of Aluminum-Carbon Nanotube Composites and Their Electrical Properties. Carbon.

[33] Ulloa-Castillo N.A., Martínez-Romero O., Hernandez-Maya R., Segura-Cárdenas E., Elías-Zúñiga A. (2021). Spark plasma sintering of aluminum-based powders reinforced with carbon nanotubes: Investigation of electrical conductivity and hardness properties. Materials.

[34] Mote V., Purushotham Y., Dole B. (2012). Williamson-Hall analysis in estimation of lattice strain in nanometer-sized ZnO particles. J. Theor. Appl. Phys..

[35] Xiao G.H., Tao N.R., Lu K. (2008). Effects of strain, strain rate and temperature on deformation twinning in a Cu–Zn alloy. Scr. Mater..

[36] Shin S.E., Bae D.H. (2013). Strengthening behavior of chopped multi-walled carbon nanotube reinforced aluminum matrix composites. Mater. Charact..

[37] Zhbanov A.I., Pogorelov E.G., Chang Y.C. (2010). Van der Waals interaction between two crossed carbon nanotubes. ACS Nano.

[38] Zare Y., Yop Rhee K. (2020). Calculation of the electrical conductivity of polymer nanocomposites assuming the interphase layer surrounding carbon nanotubes. Polymers.

[39] Zare Y., Yop Rhee K., Park S.J. (2009). Modeling the roles of carbon nanotubes and interphase dimensions in the conductivity of nanocomposites. Results Phys..

[40] Chen B., Kondoh K. (2016). Sintering behaviors of carbon nanotubes-aluminum composite powders. Metals.

